# Supplementary material for: Dynalign II: common secondary structure prediction for RNA homologs with domain insertions
Source: Nucleic Acids Res. 2014 Nov 21;42(22):13939–48. doi: 10.1093/nar/gku1172 (PMC4267632; doi:10.1093/nar/gku1172)
Supplement: SUPPLEMENTARY DATA [file supp_gku1172_nar-02021-z-2014-File012.zip › manual/RNA_class/html/functions_func_0x72.html]

RNAstructure Classes: Class Members - Functions

- Main Page
- Classes
- Files

- Class List
- Class Hierarchy
- Class Members

- All
- Functions
- Variables
- Typedefs

- a
- b
- c
- d
- e
- f
- g
- h
- m
- n
- o
- p
- r
- s
- t
- v
- w
- ~

### - r -

- Randomize()
  : Multilign\_object- ReadAlignmentConstraints()
    : Dynalign\_object- ReadConstraints()
      : RNA- ReadDSO()
        : RNA- ReadExperimentalPairBonus()
          : RNA- ReadSHAPE()
            : RNA- ReadSSO()
              : RNA- ReadThermodynamic()
                : Thermodynamics- ReFoldSingleStrand()
                  : RNA- RemoveBasePair()
                    : RNA- RemoveConstraints()
                      : RNA- RemoveOneInput()
                        : Multilign\_object- RemovePairs()
                          : RNA- RNA()
                            : RNA

---

Generated on Tue Jul 30 10:52:29 2013 for RNAstructure Classes by 
 1.5.7.1
